# Happy life expectancy among older adults: differences by sex and functional limitations

**DOI:** 10.1590/S1518-8787.2016050006727

**Published:** 2016-10-26

**Authors:** Margareth G Lima, Ana Paula Belon, Marilisa BA Barros

**Affiliations:** I Departamento de Saúde Coletiva. Faculdade de Ciências Médicas. Universidade Estadual de Campinas. Campinas, SP, Brasil; II School of Public Health. University of Alberta. Alberta, Canada

**Keywords:** Older adults, Happiness, Life Expectancy, Functional Disability, Quality of Life, Gender and Health

## Abstract

**OBJECTIVE:**

To evaluate if the happy life expectancy in older adults differs according to sex and functional limitations.

**METHODS:**

Life expectancy was estimated by Chiang method, and happy life expectancy was estimated by Sullivan method, combining mortality data with the prevalence of happiness. The questions on happiness and limitations came from a health survey, which interviewed 1,514 non-institutionalized older adults living in the city of Campinas, SP, Southeastern Brazil. The happy life expectancy was estimated by sex, age, and functional limitations. Based on the variance and standard error of the happy life expectancy, we estimated 95% confidence intervals, which allowed us to compare the statistical differences of the number of happy years lived among men and women.

**RESULTS:**

Differences by sex in happy life expectancy were significant at ages 60, 65, and 70. In absolute terms, women live more years happily. But, in relative terms, older men could expect to live proportionally more years with happiness. Happy life expectancy decreased significantly with increasing age in both men and women. Among older people living without functional limitation, differences by sex were statistically significant in all age groups, except at age 80. In the group with limitations, no significant differences by sex were found. Significant differences between the group without and with functional limitations were seen in both men and women.

**CONCLUSIONS:**

Older men could expect to live a greater proportion of their lives happily in comparison to same-aged women, but women show more years with happiness than men. Functional limitations have a significant impact on happy life expectancy for both sexes.

## INTRODUCTION

Life expectancy (LE) has improved substantially since the past century, thanks to better living and health conditions^8^. In less than 25 years, the global life expectancy at birth increased from 64 to 71 years between 1990 and 2013^23^. Projections show that life expectancy will continue to increase, even in developed countries with the largest human lifespan^8^. In the last decades, an important question raised in public health is whether the increase of life expectancy would lead to possible health deterioration and worsening of well-being. To answer this question, researchers have investigated the healthy life expectancy, a measure that combines life expectancy with health indicators, such as morbidities (e.g., diabetes), self-rated health, functional capacity, quality of life and well-being^19^ ^,^ ^24^.

Under this investigation of the expansion of life years, an important issue is the morbidity-mortality paradox according to sex. It is well-known that women have more chronic diseases and functional limitations and complain more about health than men^8^; however, they live longer than men^15^ ^,^ ^17^. The explanation to this paradox may be due to biological, sociocultural, and behavioral differences between men and women^15^. Given that the magnitude of health differences between men and women may vary according to the health indicator studied, some researchers have advocated for the use of different indicators, including the healthy life expectancy, to better understand the differences by sex in health^17^ ^,^ ^19^ ^,^ ^24^.

A step further taken by healthy life expectancy studies is the incorporation of the feeling of happiness, which is a well-being indicator used in several contexts of health research^2^ ^,^ ^9^. Resulted from the combination of the prevalence of happiness and mortality data, the happy life expectancy (happy LE) estimates how long people live and for how many years they live happily. Happy LE has some important particularities. First, the self-report of happiness is more subjective than the self-report of functional limitations and morbidities^2^ ^,^ ^24^. Second, it is more easily understood than quality of life (which is comprised of various meanings)^21^, summarizing the well-being in a more direct way.

Despite its advantages, a relative small number of studies have investigated happy LE by sex^19^ ^,^ ^24^ ^,^ ^a^. Their findings have pointed out a greater absolute number of happy years among women^24^ ^,^ ^a^, but higher proportion of happy life years lived among men^23^.

According to some studies, aging does not lead to a reduction of the feeling of happiness and of proportion of happy years^10^ ^,^ ^14^ ^,^ ^24^; however, it leads to losses in different health domains, such as the increase in comorbidities and reduction of both physical and cognitive capacity^7^. The functional capacity is defined as the ability to perform daily activities, including for mobility (e.g., climbing a flight of stairs) and self-care (e.g., using the toilet)^16^. It is a key health domain for ensuring the autonomy and independence of older adults, and preserving their quality of life^22^.

To our best knowledge, no Brazilian studies have examined happy LE among the Brazilian older population and have stratified it according to the presence of functional limitations. Therefore, because of the lack of knowledge on this subject up to now, this study aimed to evaluate the differences by sex in happy LE among Brazilian older adults, as well as analyze happy LE by the presence of functional limitations.

Our hypothesis is that both older men and women with no functional limitation would present higher happy LE than their respective counterparts living with one or more functional limitations, given the strong association of functional capacity on well-being^9^ ^,^ ^14^. A secondary hypothesis is that women would have higher happy LE in absolute terms, considering that (1) they live longer and (2) the feeling of happiness does not necessarily decrease with the increase of age.

## METHODS

Our study uses data from the 2008 ISACamp, which is a population-based household survey on health conditions conducted in Campinas, SP, Brazil, in the year of 2008. To estimate a prevalence of 50.0% with 95% confidence intervals, a sampling error of 4.0%-5.0%, and a design effect of two, the minimum sample size was estimated in 1,000 older adults.

This survey used a two-stage sample design. First, we selected 50 census tracts with a probability proportional to the number of households in each unit. Then, households were randomly sampled in each census tract selected. Using the 2010 census population distribution, we estimated the probability of older adults living in the households; this probability determined the number of households to be visited. The sample size was comprised of 3,900 households, already considering a possible loss of 20.0% due to refusals and closed households. In total, 1,519 older adults were interviewed.

To develop abridged life tables by sex, we used mortality data from *Sistema de Informações sobre Mortalidade* (SIM – Mortality Information System)^b^ and 2008 population data from *Fundação Sistema Estadual de Análise de Dados* (SEADE – Sao Paulo State Data Analysis System Foundation)^c^.

Participants were asked to evaluate their happiness by responding a single question: “How much of the time have you felt happy during the past four weeks?” Participants were asked to give the one answer that would come closest to the way they were feeling. We dichotomized the responses to code happiness: the answers “all the time” and “most of the time” were scored as “zero” (happy status), while “some of the time”, “a little of the time”, and “none of the time” were scored as “one” (unhappy status).

In addition to happiness, we used a measure of functional limitations. Participants were asked if they had any limitation when performing the following activities: walking more than one mile, walking several blocks, walking one block, and eating or bathing themselves. Their answers were dichotomized: without any functional limitation and with at least one functional limitation. Similar questions were used in other studies^3^ ^,^ ^5^.

We estimated age-specific mortality rates for five-year intervals from 60 years to 80 years or older, using 2007-2009 triennium mortality data and 2008 population. The mortality rates were then converted into death probabilities at exact age, which were used to obtain the life expectancy. We used Chiang method^6^ to construct abridged life tables for men and for women.

Happy LE was estimated using Sullivan method^18^, which uses the prevalence of happiness in each age group to determine the average number of life years that would be lived with and without happiness. Sullivan method divides the number of person-years (a function of the life table) into years to be lived with and without a certain health condition for each specific age group. Happy LE was calculated for each age group, sex, and functional limitation group (with or without).

This study was approved by the University of Campinas Research Ethics Committee (Process: 841.196). Informed consent was obtained from all participants.

## RESULTS

Table 1 presents the estimates of happiness prevalence rates for age groups and sex. There were no significant changes in the prevalence of happiness with age for both men and women. For each age group, no differences by sex were detected.

**Table 1:** Prevalence of happiness by sex and age. Campinas, SP, Southeastern Brazil, 2008.

Age groups	Males				Females				Differences by sex	
		
	Happiness									

	n	%^a^	95%CI	p^b^	n	%^a^	95%CI	p^b^		p^c^
60	218	82.0	74.6–87.6	0.1618	256	75.7	68.4–81.7	0.1379		0.0606
65	132	83.9	75.8–89.7		214	77.9	70.5–83.8			0.2099
70	116	75.1	66.1–82.4		164	68.9	58.6–77.6			0.3882
75	81	72.0	63.8–78.9		136	72.5	62.5–80.7			0.9204
≥ 80	66	79.2	66.2–88.1		131	66.5	55.6–73.6			0.0793

^a^ Weighted.

^b^ p-value related to age differences in each sex (at the 5% level).

^c^ p-value related to the differences between sexes in each age group (at the 5% level).

**Figure 1:**
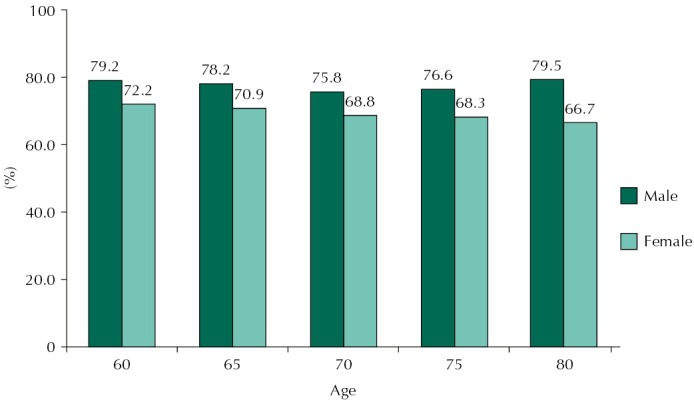
Proportion of happy life expectancy by sex and age.

The estimates of life expectancy and happy LE for men and women are shown in Table 2. Differences by sex in life expectancy were significant in all age groups; they decreased with age, from 4.5 years at age 60 to 2.0 years at age 80. For happy LE (in years), the number of happy years lived decreased with age in both sexes. Differences by sex in happy LE were significant at ages 60, 65, and 70, with women living more years happily. Men aged 60 could expect to live 15.2 years happily, while women at same age would live 17.1 years – a significant difference of 1.9 years. But, in relative terms, older men could expect to live proportionally more years with happiness in comparison to older women; this difference increased with age and was larger at age 80 (Figure 1). While the proportion of happy years showed almost no change with age among men, it decreased among women.

**Table 2:** Life expectancy and happy life expectancy by sex and age. Campinas, SP, Southeastern Brazil, 2008.

Age	Life expectancy					Happy life expectancy				
	
	Males		Females		Differences by sex	Males		Females		Differences by sex
					
	Years	95%CI	Years	95%CI		Years	95%CI	Years	95%CI	
									
	(A)		(B)		(B-A)^a^	(C)		(D)		(D-C)^a^
60	19.2	18.2–20.2	23.7	22.8–24.6	4.5^b^	15.2	14.5–15.8	17.1	16.3–17.8	1.9^b^
65	15.6	14.6–16.5	19.6	18.8–20.5	4.0^b^	12.2	11.5–12.8	13.9	13.2–14.6	1.7^b^
70	12.4	11.5–13.2	15.7	15.0–16.5	3.4^b^	9.4	8.7–10.0	10.8	10.1–11.5	1.4^b^
75	9.4	8.7–10.2	12.3	11.7–12.9	2.9^b^	7.2	6.5–7.8	8.4	7.7–9.2	1.2
80^c^	7.3		9.3		2.0	5.8	5.0–6.5	6.2	5.4–6.9	0.4

^a^ At the 5.0% level.

^b^ Statistically significant differences by sex.

^c^ Note: 95% confidence intervals for life expectancy could not be estimated for the age 80.

Regarding happy LE according to functional limitations (Table 3), the happy years gradually decrease with increasing age in men and women for older adults with and without functional limitations. Among older adults living without functional limitation, differences by sex were statistically significant in all age groups, except at age 80. In the group with at least one functional limitation, no significant differences by sex were found.

**Table 3:** Happy life expectancy by sex, according to functional disability. Campinas, SP, Southeastern Brazil, 2008.

Age	Without limitations					With limitations					Without - with limitations	
		
	Males		Females		Diferences by sex	Males		Females		Diferences by sex	Males	Females
							
	Years (A)	95%CI	Years (B)	95%CI	(B-A)^a^	Years (C)	95%CI	Years (D)	95%CI	(D-C)^a^	(A-C)^a^	(B-D)^a^
60	16.5	15.8–17.2	19.0	17.9–20.0	2.5^b^	13.2	12.0–14.4	15.7	13.8–17.5	2.5	3.3^b^	3.3^b^
65	13.5	12.8–14.2	15.8	14.7–16.8	2.3^b^	10.4	9.3–11.6	12.8	11.1–14.5	2.4	3.1^b^	3.0^b^
70	10.5	9.7–11.2	12.5	11.5–13.6	2.0^b^	8.2	7.2–9.2	10.1	8.6–11.6	1.9	2.3^b^	2.4
75	8.1	7.3–8.8	10.2	9.1–11.3	2.1^b^	6.4	5.4–7.4	7.7	6.3–9.2	1.3	1.7	2.5
≥ 80	6.8	6.1–7.4	7.9	6.7–9.0	1.1	4.9	3.8–6.0	5.6	4.2–7.0	0.7	1.8^b^	2.3

^a^ At the 5% level.

^b^ Statistically significant differences by sex.

Analyzing men and women separately, one can observe significant differences between the group without and with functional limitations. Except for those aged 75 years, men without any functional limitations could expect to live significantly more years than men with at least one functional limitation. The same pattern can be seen for women at ages 60 and 65. At age 60, men and women without functional limitations would live 3.3 years more than their counterparts living with at least one functional limitation. The difference reduced with the increase of age in both men and women.

**Figure 2:**
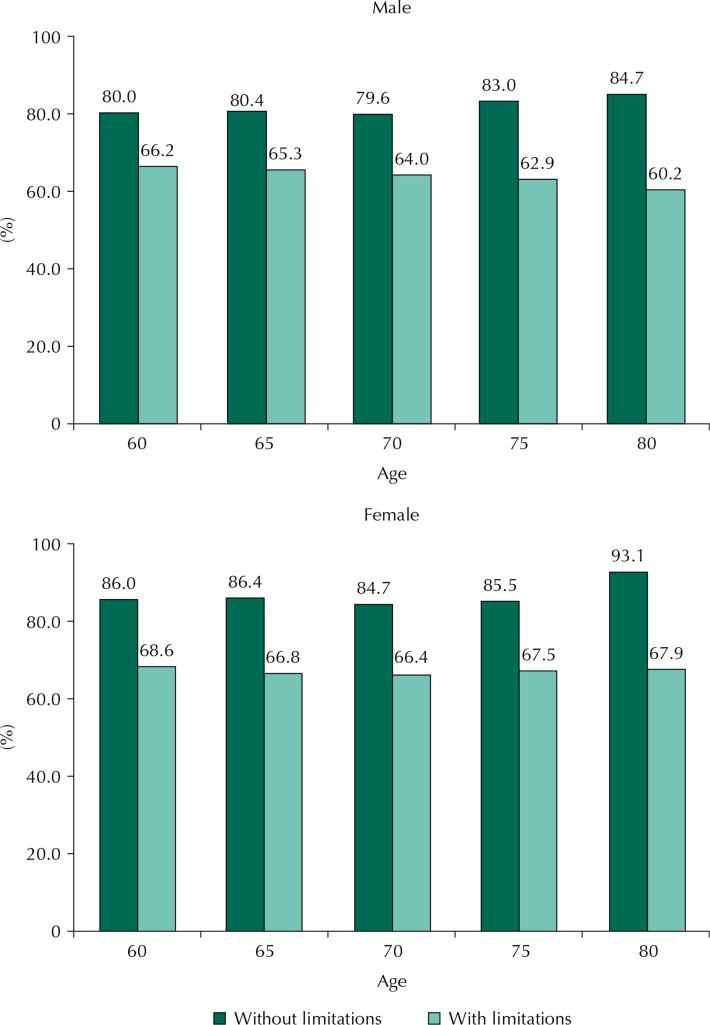
Proportion of happy life expectancy according to functional disability, by sex and age.

The proportion of years lived happily reduced considerably in the group with functional limitations in comparison to the group without functional limitations, for men and for women (Figure 2).

## DISCUSSION

Our study examined how long Brazilian older adults aged 60 years or more can expect to live happily. The estimates were disaggregated by five-year age groups and sex. The analysis was also stratified by the absence and presence of functional limitations. Happy LE resulted from the combination of life expectancy and feeling of happiness measured in the past four weeks before the survey.

With the expansion of the longevity, the research on quality of life years gained and sex inequalities on health and mortality has made important progress thanks to estimation of healthy life expectancy^17^. Studies have calculated healthy life expectancy in combination with specific diseases, functional limitations^1^ ^,^ ^5^, and self-rated health^3^, which can be interpreted as indirect measures of well-being and quality of life^4^. However, studies on happy LE are still emerging in the literature. Most studies^15^ ^,^ ^20^ ^,^ ^24^ have compared the number of happy years among countries and have used data from the World Database of Happiness project, which has compiled surveys on happiness worldwide since the post-war period^20^. Little work has been done to estimate happy LE in Latin America, and more specifically in Brazil. Our study may be the first one analyzing differences by sex in happy LE among Brazilian older adults.

Consistent with other studies^10^ ^,^ ^14^, no differences by sex in the prevalence rate of happiness were found; however, they did exist in the happy LE. At ages 60, 65, and 70, the number of happy years expected to be lived were significantly higher among women, while the proportion of happy years is greater among men. These findings confirm a previous research showing that women from the five countries studied would live more years in total and happily than men^a^. A study analyzing the United States trends and patterns of happy LE between 1970 and 2000 found similar results: in comparison to men, happy LE estimated for women was greater in absolute terms, but smaller in relative terms^24^. However, while this study showed no change with age in the proportions of happy LE among older men and women, we found that the proportion of happy life years decreases with age in the female population, but it does not change in the male population.

Using data from 45 countries, a study compared differences by sex in healthy life expectancy with several morbidity and functional limitation indicators. The results showed a female advantage in terms of life expectancy (on average, 6.3 years higher than male life expectancy) and healthy life expectancy, in absolute and relative terms^15^. Based on these findings, the authors suggested that women have a higher healthy life expectancy because they have a longer life expectancy. However, findings from different studies on differences by sex in healthy life expectancy are divergent, when indicators of functional limitations and self-rated health were used. Some studies have shown that women could expect to live higher proportion of their lives in good health^3^, while others found the opposite^1^ ^,^ ^13^.

Previous studies have also detected that the female or male advantage in the healthy life expectancy depends on the health indicator used^3^ and level of mortality of the countries^19^. Belon et al.^3^ found different results according to the health indicator studied: older Brazilian women could expect to live longer and most of the years would be spent with better self-rated health. However, they would live more years with functional limitations in contrast to older Brazilian men. Using data on disability prevalence from 25 European countries, a study on healthy life expectancy showed that in populations with high life expectancy women could expect to live more unhealthy years than men; in contrast, in countries with low life expectancy men could expect to live a shorter life expectancy and longer unhealthy life expectancy with poorer health^19^.

Our study also estimated the happy LE according to the presence of functional limitations. Our research, performed with the same population of this study, showed that functional limitation is significantly higher among women than men who are aged 80 years or older, and functional limitation tends to increase with age in both sexes^3^.

To our best knowledge, this is the first study that analyzed these conditions in combination with happiness. In comparison to the absence of functional limitations, the male and female happy LE reduced significantly in both absolute and relative terms. Other studies showed a strong association between functional limitations and happiness^9^ ^,^ ^14^. In our previous study, with the same Brazilian sample, we found an association between happiness and functional limitations; the prevalence of happiness was 2.5 times higher among older adults without than with functional limitations^14^. Examining the relationship of life satisfaction and perception of future happiness with functional status, an eight-year cohort study with 3,363 people found that well-being is a protective factor from increased mobility limitations^9^.

Regarding the older adults who did not report the functional limitations studied, happy LE statistically differed between sexes, with a female advantage. However, among older adults who reported one or more functional limitations, these differences were not significant. These findings suggest that functional limitations may have a substantial negative impact on the happiness levels of older adults. Therefore, the promotion of an active aging with more years free of functional limitations coupled with compensatory strategies supporting autonomy and independence among older adults are fundamental for their quality of life and happiness^22^. According to Van Oyen et al.^19^, the higher prevalence of functional limitations among women is responsible for decreasing the differences by sex in healthy life expectancy, which otherwise would be greater.

The limitations of this study are related to the question about happiness used. Although this question is part of an instrument that assesses health-related quality of life (i.e., SF-36) and is not meant to be used separately, it is still a sensible, valid indicative of happiness. First, because the question considers the frequency and duration of happiness, which has been considered a better indicative of well-being in comparison to the intensity of happiness^12^. Second, because it is efficient to measure happiness at population level^11^. Another limitation of this study is that institutionalized Brazilian older adults were not included. Although less than 1.0% of the total older population lives in long-term care facilities^d^, the generalizability of our findings may be limited.

On the other hand, this study has several strengths. First, it adds some evidence to the limited international literature on happy LE, especially on differences by sex in happy life years in the presence of functional limitations. Second, to our best knowledge, this is the first study analyzing differences by sex in happy LE using data from Brazilian surveys. Third, this study is among the first ones that estimate 95% confidence intervals of happy LE, which enables us to analyze the statistical significance when comparing results.

Our research found that older women would live longer than men and with more happy years. However, older men could expect to live a greater proportion of their lives happily in comparison to same-aged women. Happy LE decreased significantly with age among older men and women, including among those who reported no or at least one functional limitation. No difference by sex among older adults with functional limitations was detected. The proportion of happy LE did not increase with age among men and women with no functional limitations, although it was relatively larger at age 80 in both sexes. Among men and women who have at least one functional limitation, the proportion of happy LE decreased with age.

Given our findings, it is clear the relevance of strategies that help maintain older adults’ autonomy and independence, which are essential to their well-being and feeling of happiness. More importantly, our study suggests that if men had a longer life expectancy than women, they could expect to live more years happily than women. Thereby, health research and public policies should consider the differences by sex, besides the quantity of years to be lived and quality of life.

## Footnotes

a Kunst AE, Okma-Keulen PT, Veenhoven R. Happy life expectancy in 5 European countries. XIII World Congress of Sociology Bielefeld, Germany 1994 [cited 2016 Sept 13]. Available from: https://personal.eur.nl/veenhoven/Pub1990s/94f-full.pdf

b Ministério da Saúde. Sistema de Informações sobre Mortalidade – SIM . Available from: http://svs.aids.gov.br/cgiae/sim/

c Fundação Sistema Estadual de Análise de Dados – SEADE. Available from: http://www.seade.gov.br/

d Instituto de Pesquisa Econômica Aplicada (IPEA).Condições de funcionamento e infraestrutura das instituições de longa permanência para idosos no Brasil. Brasília (DF): IPEA; 2011 (Comunicado do IPEA, Vol 93).
